# Effects of Psychological Capital and Person-Job Fit on Hospitality Employees’ Work-Family Conflict, Family-Work Conflict and Job Performance: The Moderating Role of Marital Status

**DOI:** 10.3389/fpsyg.2022.868971

**Published:** 2022-05-06

**Authors:** Zhen Yan, Na Bai, Zuraina Dato Mansor, Wei Chong Choo

**Affiliations:** ^1^Faculty of Hotel Management, Qingdao Vocational and Technical College of Hotel Management, Qingdao, China; ^2^Graduate School of Business, Universiti Sains Malaysia, Pulau Pinang, Malaysia; ^3^School of Business and Economics, Universiti Putra Malaysia, Serdang, Malaysia

**Keywords:** psychological capital, person-job fit, work-family conflict, family work conflict, job performance, marital status

## Abstract

Drawing on the conservation of resources (COR) theory and congruence theory, this study aims to investigate the influence of psychological capital (PsyCap) and person-job fit (PJ fit) on work–family conflict (WFC), family–work conflict (FWC) and job performance (JP), especially the moderating effect of marital status on hypothesized relationships between two directions of conflicts in the work-family interface and JP. Utilizing a two-stage design, this study surveyed 312 flight attendants employed by two international airline companies in Malaysia and used the structural equation modeling technique to test the hypothesized relationships. Findings showed that PsyCap could significantly alleviate two directions of WFC simultaneously and promote employees’ JP. PJ fit was also identified to be an effective mitigator of WFC and FWC; however, a significant association between PJ fit and JP has not been found in this study. The findings further suggested that both WFC and FWC could mediate the association between PsyCap and JP. In addition, the fact that marital status resulted in disparity in the formation of JP was also evidenced. Airline companies should pay more attention to the positive impact of individual psychological determinants, such as PsyCap and PJ fit, which can effectively alleviate various issues in the work–family interface, thereby improving employees’ JP.

## Introduction

In 2019, the hospitality industry accounted for approximately 10.4% of global GDP (USD 9.2 trillion); more importantly, it provided 10.6% of all jobs (334 million) (World Travel and Tourism Council, 2020). Later, due to the impact of the coronavirus 2019 (COVID-19) pandemic, 62 million jobs were lost, and it was predicted that the lost jobs could be returned before the end of 2022 (World Travel and Tourism Council, 2020). Given the size of workforce, both researchers and practitioners should be concerned with identifying and breaking down obstacles that may arise in the workplace. In fact, unlike any other industry, the hospitality industry contains organizations that often operate 24 h a day and 7 days a week (O’Neill and Follmer, 2020). As a result, such organizations require employees to invest large amounts of time and assume more responsibilities, which can make it tough for hospitality employees to achieve work-life balance (Sun et al., 2020). While the conflict between work and family can be a challenge for many employees regardless of industry, it can be especially harmful for hospitality employees on the account of their irregular working schedules, long working hours, and high intensity of customer service (Zhao and Ghiselli, 2016; Ilkhanizadeh and Karatepe, 2018; Schiffinger and Braun, 2020; Shin et al., 2021).

In general, work-family conflict (WFC) and family work conflict (FWC) occur when employees fail to balance the demands of work and family roles (Charoensukmongkol and Puyod, 2021). Two directions of conflicts in the work–family interface always result in undesirable work-related outcomes. For examples, previous studies have found that WFC and/or FWC could hinder job performance (JP) (e.g., Li et al., 2017; Soomro et al., 2018). Over the past decades, a great deal of attention has been paid to employees’ JP, especially in the service industry. Organizational performance is mainly influenced by individual performance, which is the foundation of organizational profitability and success (Almatrooshi et al., 2016; Cooper et al., 2019). For a long time, the management of organizations has been trying to constantly improve the performance of employees (Dresner and Howard, 2007).

The central principle of the conservation of resources (COR) theory is that people strive to acquire, retain, and protect resources (Hobfoll, 1989a). Resources are defined as objects (e.g., house and tool), personal characteristics (e.g., PsyCap), conditions (e.g., organizational support), or energies (e.g., time, physical/mental energy) (Agarwal and Avey, 2020; Charoensukmongkol and Puyod, 2020; Charoensukmongkol, 2021). In light of the COR theory, both psychological capital (PsyCap) and person-job fit (PJ fit) are considered as personal key resources (Wheeler and Halbesleben, 2009; Avey et al., 2010). Therefore, PsyCap and PJ fit are supposed to mitigate two directions of work–family conflict and promote employees’ JP in the workplace. According to the congruence theory, the similarity between work and family can be created by a third variable as a common cause, and that this aforementioned third variable includes personality variables, social and cultural forces, and so on (Edwards and Rothbard, 2000). Because both PsyCap and PJ fit are personality variables, they can reduce individuals’ perceptions of conflicts in the work–family interface. That is to say, employees’ PsyCap and PJ fit can mitigate their conflicts in the work–family interface effectively.

Although many researchers have explored the impact of personal psychological resources (such as PsyCap and PJ fit) on hospitality employees’ emotions, attitudes, and behaviors in recent years (Kim et al., 2017; Han and Hwang, 2019; Ozturk and Karatepe, 2019; Khliefat et al., 2021; Saleem et al., 2021; Yan et al., 2021b), extant studies on these subjects have obvious limitations. First, findings of the effect of PsyCap on the two directions, WFC and FWC, are still scarce, especially in the hospitality sector (Karatepe and Karadas, 2014). Second, to our knowledge, no study has ever investigated how PJ fit affects WFC and FWC in the hospitality industry except for Karatepe and Karadas (2016). Third, findings on the effect of PJ fit on job performance are inconsistent. Many previous studies have concluded that PJ fit positively influenced JP (Lin et al., 2014; Iqbal et al., 2020; Ma et al., 2021). However, Kristof-Brown et al.’s (2005) meta-analysis showed that PJ fit only had a modest correlation with JP (*r* = 0.2). In addition, several studies noted that PJ fit was not significantly related to JP (Lauver and Kristof-Brown, 2001; Greguras and Diefendorff, 2009). That is to say, the relationship between PJ fit and JP is not that steady.

Factors that might moderate the effects of WFC and FWC remain unclear. According to Greenhaus and Beutell (1985), WFC or FWC referred to a role conflict caused by undertaking too many responsibilities at work and at home. WFC and FWC are two stressors in the work–family interface (Karatepe and Karadas, 2014). Such two conflicts always lead to undesirable work-related outcomes, such as job stress, burnout, task distraction, reduced organizational commitment and job performance (Mansour and Tremblay, 2018; Smith et al., 2018; Wu et al., 2018). According to the COR theory, a marital relationship can be viewed as a valued resource (Grandey and Cropanzano, 1999). Those who are married and living together may have more resources to draw on (i.e., their spouse and more finances), than those who are not living with someone in a committed relationship. They are supposed to have lower levels of stress (Grandey and Cropanzano, 1999) and may invest more time and energy to promote their job performance (Hou et al., 2020). On the other hand, married employees are often believed to have more family role responsibilities (Michel et al., 2011). In this case, marital status might moderate the relationship among WFC, FWC, and JP.

Despite research having investigated PsyCap in North America (e.g., Abbas et al., 2014; Choi et al., 2014) and other European countries (e.g., Ozturk and Karatepe, 2019; Sesen and Ertan, 2019), there is limited knowledge of the effect of PsyCap on the Asian hospitality industry. Nolzen (2018) also stressed that the extension of research stream to other social systems or cultures is critical for the validity and applicability of the PsyCap concept.

To fill these research gaps, we proposed and tested a research model to deal with the question of whether PsyCap and PJ fit were linked to JP *via* WFC and FWC, and whether marital status resulted in disparity in the formation of JP. Such relationships were examined based on data obtained from flight attendants who currently work in two international airline companies in Malaysia. Finally, the results of this research will have beneficial implications in the improvement of hospitality employees’ JP as well as management of their work and family roles.

## Literature Review and Hypotheses Development

### Conservation of Resources Theory and Congruence Theory

Hobfoll (1989b) proposes that the main principle of the COR theory is that people tend to acquire, retain, protect, and cultivate resources that they cherish. Avey et al. (2010) point out that resilience, hope, self-efficacy, and optimism are four dimensions of PsyCap and personal resources within the scope of the COR theory. Kim and Hyun (2017) highlight that PsyCap’s four components are key resources for employees, enabling them to adapt to adversity, solve difficulties, and perform well in the workplace. Hobfoll (2001) classifies 74 resources into four general categories: (a) personal (KSAs, traits, etc.), (b) condition (such as status), (c) object (such as material assets and compensation), and (d) energy (such as time and effort). Based on the conceptualization of PJ fit (e.g., demand-ability fit, need-supply fit), fit could exist as any of these four categories of resources. Therefore, PJ fit can also be regarded as a personal resource based on the COR theory (Wheeler and Halbesleben, 2009).

In light of the COR theory, staff who possess high PsyCap and PJ fit can deal with problems and difficulties that arise from both family and work domains (Karatepe and Karadas, 2014; Islam et al., 2019) and show higher JP accordingly (Avey et al., 2010; Guido et al., 2018). First of all, Hobfoll (2002) believed that staff with key resources tended to be more capable of selecting, changing, and implementing their other types of resources to satisfy stressful demands. To be specific, such employees are able to invest their personal resources so that they can manage WFC or FWC and recover from losses. As a matter of fact, they have established a balance between their family and work roles. Nevertheless, if employees do not have sufficient personal resources, they will not be able to handle various issues in the work–family interface. Second, Avey et al.’s (2011) meta-analysis demonstrated that employees with high levels of PsyCap were more satisfied with their work and performed better. PsyCap helps employees maintain an internalized sense of control and intent that is necessary for them to achieve targets or meet job requirements (Choi et al., 2019). Moreover, employees with high levels of PJ fit are more likely to complete their jobs punctually and meet performance expectations successfully (Werbel and DeMarie, 2005; Ma et al., 2021).

**FIGURE 1:**
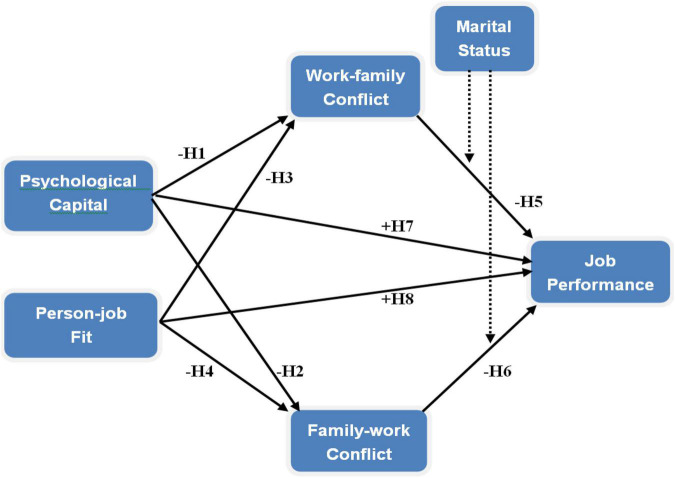
Conceptual model.

From the perspective of the congruence theory, it can also offer guidance for developing the association between personality variables and conflicts in the work–family interface. According to Edwards and Rothbard (2000), congruence refers to a type of similarity between employees’ work and family. Edwards and Rothbard (2000) stated that the congruence theory attributed this similarity to a third variable that affects the work and family domains. Edwards and Rothbard (2000) further explained that the third variable was a common cause composed of social and cultural influence, personality variables, and general behavioral style. In light of Michel et al. (2011), the personality variable may be the third variable, which has a dual influence on employees’ cognition of WFC and FWC. A careful review of the extant literature also reveals the direct influence of personality variables (e.g., self-efficacy and PJ fit) on both WFC and FWC (Allen et al., 2012; Karatepe and Karadas, 2016). Accordingly, this study proposes that employees’ PsyCap and PJ fit influence their WFC and FWC, thus providing adequate control over the inter-play between work and family roles. Thus, the conceptual model was proposed (see Figure 1).

### Psychological Capital, Person-Job Fit, and Conflicts in the Work–Family Interface

The concept of PsyCap was originally proposed by Luthans and Youssef (2004). PsyCap, which is manifested by four key components, resilience (when hindered by adversity or difficulties, sustaining and bouncing back to achieve goals), self-efficacy (having confidence to exert great efforts to complete challenging missions), hope (pursing targets, if necessary, redirecting ways to targets so as to succeed), and optimism (making positive attributions for present and future success) is a relatively new psychological construct in organizational behavior (Luthans and Youssef, 2004). PJ fit is defined as the degree of consistency between employees’ personal characteristics and job characteristics. It can be further divided into the degree to which an individual’s ability fits the demands of work (demand-ability fit) and the degree to which individual’s needs fit what job supplied (need-supply fit) (Kristof-Brown et al., 2005). When employees have rich knowledge, exquisite technology, and strong personal ability that meet work requirements, it is demand-ability fit; when work meets the expectation and demand of employees, it is need-supply fit (Cable and DeRue, 2002). According to Greenhaus and Beutell (1985), WFC referred to a role conflict caused by undertaking too many responsibilities at work and at home. Besides, researchers revealed that WFC was generated by bidirectional effects, namely, WFC and FWC (Gutek et al., 1991). WFC and FWC are two stressors in the work–family interface (Karatepe and Karadas, 2014). Since flight attendants are often confronted with WFC and FWC because of uncertain work schedules and regular overtime work, effectively managing work and family roles has become quite important for them (Soomro et al., 2018; Aboobaker and Edward, 2020). This study highlighted that PsyCap and PJ fit as two important personal resources could prevent employees from suffering conflicts in the work–family interface based on the COR theory. Nevertheless, very little is known about the simultaneous effects of PsyCap and PJ fit on both directions of conflict in a study. Thus, based on the above, the following hypotheses are put forward:

H1: PsyCap is negatively correlated with WFC,
H2: PsyCap is negatively correlated with FWC,
H3: PJ fit is negatively correlated with WFC, and
H4: PJ fit is negatively correlated with FWC.

### Conflicts in the Work–Family Interface and Job Performance

Darvishmotevali and Ali (2020) stated that employees’ JP in an organization reflects their ethical values, knowledge, skills, and behavior. It is also the employees’ ability to meet organizational expectations. The COR theory provides guidance on the association between conflicts in the work–family interface and job outcomes like JP. Shaffer et al. (2001) also stressed that excessive demands and/or inadequate personal resources within a specific role domain or between domains could lead to negative emotions and dysfunctional behaviors. According to the COR theory, these frontline employees tend to conserve their scarce resources through intention to leave their organizations, which can have a negative impact on JP to a great extent, as they realize that they may not be able to deal with problems arising from both WFC and FWC (Mansour and Tremblay, 2018). Accordingly, they are hypothesized that:

H5: WFC is negatively correlated with JP.
H6: FWC is negatively correlated with JP.

### Psychological Capital, Person-Job Fit, and Job Performance

Person-job fit, especially DA fit, is relevant to an individual’s compatibility with a specific job (Kristof-Brown, 2000). Employees with knowledge, skills, and abilities (KSAs) that match job requirements are expected to perform their job effectively (Kristof-Brown, 2000). Therefore, it can be predicted that employees with high PJ fit will have better job performance than those with low PJ fit (Li and Hung, 2010). In addition, from the perspective of the COR theory, both PsyCap and PJ fit are personal resources (Wheeler and Halbesleben, 2009; Avey et al., 2010), and the accumulation of personal resources is indispensable for goal pursuit (Story et al., 2013). Individuals with sufficient resources have confidence in completing tasks successfully and persevere in the face of obstacles and challenges (Hobfoll et al., 2018). Avey et al.’s (2011) meta-analysis demonstrated that individuals with high levels of PsyCap always performed better. PsyCap helps individuals maintain an internalized sense of control and intent, which is necessary to achieve desired goals or meet and exceed job requirements. Rabenu et al. (2017) also highlighted that PsyCap could promote efforts to succeed and achieve goals, which in turn lead to better performance. Lin et al. (2014) reported that PJ fit had positive effects on JP. However, Kristof-Brown et al.’s (2005) meta-analysis showed that PJ fit only had a modest correlation with JP. So far, no consensus has been reached. Thus, the following hypotheses are proposed:

H7: PsyCap is positively correlated with JP, and
H8: PJ fit is positively correlated with JP.

### Psychological Capital, Person-Job Fit, Conflicts in the Work–Family Interface, and Job Performance

According to the congruence theory, a similarity between work and family can be generated by a third variable that acts as a common cause (Edwards and Rothbard, 2000). The aforementioned third variable includes personality variables, social and cultural forces, and so on (Edwards and Rothbard, 2000). Because both PsyCap and PJ fit are personality variables, they can reduce individuals’ perceptions of conflicts in the work–family interface. That is, employees’ PsyCap and PJ fit mitigate their conflicts in the work–family interface. Under these circumstances, employees will be able to cope with difficulties and adversity arising from the management of work and family roles and, therefore, will report improved JP. There are limited empirical studies that link personality variables to outcomes *via* WFC and FWC. For example, Karatepe and Karadas (2014) reported that PsyCap was negatively related to turnover and absence intentions through WFC and FWC. The Braunstein-Bercovitz et al. (2012) research revealed that the relationship between personality variables (e.g., resilience) and well-being was partially mediated by WFC and FWC. Lestari (2020) reported that PJ fit had a negative impact on both WFC and FWC. In addition, FWC mediated the relationship between PJ fit and emotional exhaustion. However, WFC had no effect on emotional exhaustion. Basically, these findings are consistent with the argument that personality variables can be used to relieve the two directions of conflict and, thus, contribute to positive outcomes. Accordingly, the following hypotheses are advanced:

H9: the relationship between PsyCap and JP is mediated by WFC,
H10: the relationship between PsyCap and JP is mediated by FWC,
H11: the relationship between PJ fit and JP is mediated by WFC, and
H12: the relationship between PJ fit and JP is mediated by FWC.

### Marital Status, Work–Family Conflict, Family–Work Conflict, and Job Performance

As stated in the COR theory, marital status can be viewed as a valued resource (Grandey and Cropanzano, 1999). Those who are married and living together may have more resources to draw on (i.e., their spouse and more finances) than those who are not living with someone in a committed relationship. Those who are married/living together should have lower levels of stress (Grandey and Cropanzano, 1999). On the other hand, married employees are often believed to have more family role responsibilities (Michel et al., 2011). Accordingly, when confronted with antecedents, such as working time demands or other work domain stressors, such employees are more likely to view these antecedents as conflicts with their family lives than employees who are single (Michel et al., 2011). Byron (2005) meta-analytic review combined the results of more than 60 previous related studies and demonstrated that marital status was related to WFC and FWC. However, Allen et al.’s (2012) meta-analysis concluded that marital status was not correlated with WFC or FWC. Echoing the research of Allen et al. (2012), Amazue and Onyishi (2016) also supported that marital status was not related to WFC. In addition, Salem et al. (2013) revealed that academic performance was significantly affected by marital status. To be specific, married students were observed to have a significantly higher CGPA than single students. Thus, the following hypotheses are developed:

H13: the relationship between WFC on JP is moderated by marital status, and
H14: the relationship between FWC on JP is moderated by marital status.

## Research Methodology

### Procedure and Participants

Self-administered questionnaires were utilized to collect data from two international airline companies, namely, Air Asia and Malaysia Airlines System Berhad (MAB) in Kuala Lumpur. Given that our researchers have a good friendship with the managers of the airlines mentioned above, convenience sampling was applied in the survey. We invited cabin managers to participate in the survey. With their permission, an online survey link was randomly delivered to every potential respondent because of the serious situation of the COVID-19 pandemic. We suggested that respondents should fill out the survey with honest answers. In addition, it was stressed that we were collecting data for research purposes only, and that the confidentiality and anonymity of the survey could be guaranteed (Podsakoff et al., 2003). The questionnaire is originally written in English and in order to avoid errors and ensure validity, a scholar who is skilled at both Malay and English is responsible for translating and back-translating questionnaire (Brislin, 1980a). A panel of professional airline management was invited to examine the content validity of the questionnaire.

Data were collected with a time lag of 3 weeks in two waves (Podsakoff et al., 2012), which started on 11 October 2021. Participants who took part in the first-stage survey (time 1) were also required to participate in the second stage (Time 2). During the first-stage survey (time 1), the respondents were requested to rate the measures of PsyCap, PJ fit, WFC, and FWC and fill in personal demographic profiles. During the second-stage survey (time 2), the respondents completed the questionnaire on job performance. The researchers distributed 420 questionnaires in time I and 326 questionnaires in time 2. Finally, 317 questionnaires were returned. After checking outliers *via* the Mahalanobis distance (De Maesschalck et al., 2000), five questionnaires were removed. Finally, a total of 312 valid questionnaires were obtained.

Table 1 demonstrates the demographic characteristics of the respondents in terms of gender, age, marriage, and education. Among the participants, there were 120 (38.5%) male and 192 (61.5%) female respondents. 117 (37.5%) respondents were unmarried and 195 (62.5%) respondents were married. In terms of age, 14.3% respondents were in the age group of 18 to 24, 45.3% were 25 to 34, 35.5% were 35 to 44, while the age group of 45 and above contributed 4.9% respondents to the study. With regard to the highest level of education, 2.6% respondents achieved Master’s degree and above, 26.9% attained a Bachelor’s degree or an Associate’s degree, 33% achieved a diploma, while respondents with SPM accounted for 37.5%.

**TABLE 1:** Profile of the respondents.

Demographic Categories	Frequency (n)	Percentage (%)
**Gender**		
Male	120	38.5
Female	192	61.5
**Age (years)**		
18-24	45	14.3
25-34	141	45.3
35-44	111	35.5
45 and above	15	4.9
**Marriage**		
Unmarried	117	37.5
Married	195	62.5
**Education**		
SPM	117	37.5
Diploma	103	33.0
Bachelor’s degree/Associate’s degree	84	26.9
Master’s degree and above	8	2.6
Total	312	100

### Measures

Based on the following four measurement tools, a total of 44 items were included in the initial model. All the scales that we used are widely used research tools, with adjustments for this study. A linguist was responsible for translating and back-translating the questionnaire (Brislin, 1980b).

#### Psychological Capital

A tool with 24 items proposed by Luthans et al. (2007) has been used to appraise PsyCap. This scale consists of four dimensions, and each dimension includes six items. Responses would be recorded on a Likert six-point scale. Responses to items in PsyCap were recorded on a Likert six-point scale ranging from 1 (strongly disagree) to 6 (strongly agree). A sample item is “If I should find myself in a jam at work, I could think of many ways to get out of it.” In addition, to reduce the complexity of the structural model, the item parceling technique was used for PsyCap (Matsunaga, 2008). Thus, in this study, Cronbach’s alpha for the four dimensions were 0.75, 0.85, 0.79, and 0.77, respectively.

#### Person-Job Fit

The measurement of PJ fit is based on the two-dimensional scale developed by Cable and DeRue (2002). The two dimensions are need-supply fit (N-S fit) and demand-ability fit (D-A fit), and each dimension contains 3 questions. A sample item is “The attributes that I look for in a job are fulfilled very well by my present job.” A 7-point Likert scale is adopted, with 1 indicating strongly disagree and 7 indicating strongly agree. Cronbach’s alpha for this scale was 0.88.

#### Work–Family Conflict and Family–Work Conflict

The two directions of work-family conflicts were measured with eight items from Grzywacz and Marks (2000). We used a 5-point Likert scales ranging from 1 (never) to 5 (all the time). A sample item for WFC is “Stress at work makes you irritable at home.,” while a sample item for FWC is “Your home life helps you relax and feel ready for the next day’s work.” Cronbach’s alpha for the two scales were 0.86 and 0.87.

#### Job Performance

A 6-item Likert scale was utilized, and has been employed many times when investigating employees in the service industry (Kim et al., 2009; Chiang and Hsieh, 2012). A sample item is “I am effective in my job.” We used the seven-point Likert scale ranging from one (strongly disagree) to seven (strongly agree). Cronbach’s alpha for this scale was 0.88.

#### Control Variables

We included employee demographic characteristics (e.g., gender, age, marital status, and educational background) as controls for outcome. Previous studies have suggested that JP may vary across gender, age, marital status, and educational background (Byron, 2005; Amazue and Onyishi, 2016); thus, the above-mentioned variables were included as control variables.

### Data Analytic Strategy

Descriptive statistics and reliability tests of the research variables were conducted with SPSS 24.0. Amos was employed to conduct structural equation modeling (SEM) and test the proposed model and hypothesized relationships (Anderson and Gerbing, 1988). The measurement model was first estimated by confirmatory factor analysis (CFA), and a structural model was then analyzed for model evaluation and research hypotheses testing. In this study, after the reliability test, domain-representative parceling was conducted to create four parcels by computing the average item scores of four indicators of the PsyCap construct (Hall et al., 1999; Bandalos and Finney, 2001). Conducting item parceling (i.e., summated items produced by averaging item scores) helps to considerably reduce the number of free parameters, which makes the estimation reliable without increasing the sample size (Bagozzi and Edwards, 1998). Bandalos (2002) also stressed that item parceling minimizes non-normality issues of individual items and improves the ratio between sample size and parameters by decreasing parameters that need to be estimated. Item parcels were generated based on the subscale of each construct. In addition, the item parceling technique also reduces the complexity of structural models; thus, it was used in this study.

## Results

### Full Measurement Estimation

The full measurement model showed a satisfactory fit with the data. As we can see in Table 2, all standardized factor loadings ranged from 0.646 to 0.849, meaning that all the items could measure their corresponding constructs effectively. In addition, all the constructs’ Cronbach alpha coefficients were larger than 0.7 (Hair, 2009), which implied that all the items could reflect corresponding constructs well. As for composite reliability values for all the constructs, they were all larger than the minimum threshold of 0.7 (Hulland, 1999). What is more, all latent variables’ AVE values were larger than the threshold value of 0.5 (Bagozzi and Yi, 1988). In brief, the above findings also indicated that all the indicators measuring constructs were one-dimensional in the full measurement model. In addition, fit indices strongly supported the hypothesized full measurement model, for instance, normed X^2^ = 1.802; IFI = 0.95; RMSEA = 0.05; TLI = 0.94;CFI = 0.95, and AIC = 599.99 (Steiger, 2007; Hair, 2009).

**TABLE 2:** Results of reliability and convergent validity of the full measurement model.

Variables and items	Std. factor loading	C.R	AVE	Cronbachα
PsyCap:		0.8303	0.5504	0.829
SEv	0.767			
HOv	0.741			
OPv	0.755			
REv	0.703			
WFC:		0.8613	0.6089	0.860
WFC1	0.799			
WFC2	0.826			
WFC3	0.714			
WFC4	0.778			
FWC:		0.87	0.6266	0.868
FWC1	0.719			
FWC2	0.829			
FWC3	0.832			
FWC4	0.781			
JP:		0.8826	0.5567	0.882
JP1	0.699			
JP2	0.788			
JP3	0.787			
JP4	0.759			
JP5	0.693			
JP6	0.745			
PJ Fit:		0.8848	0.5631	0.883
PJ1	0.646			
PJ2	0.849			
PJ3	0.753			
PJ4	0.756			
PJ5	0.725			
PJ6	0.759			

In this study, discriminant validity was examined with the following method. As shown in Table 3, all the constructs’ AVE values are larger than their squared correlation coefficients. Hence, strong proof of the discriminant validity for all the constructs was offered (Henseler et al., 2015).

**TABLE 3:** Results of the discriminant validity test.

Variables	1	2	3	4	5
(1) PsyCap	***0.55***				
(2) PJ fit	0.10***	***0.56***			
(3) WFC	0.15***	0.11***	***0.61***		
(4) FWC	0.22***	0.10***	0.07***	***0.63***	
(5) JP	0.38***	0.13***	0.19***	0.21***	***0.56***

PsyCap, psychological capital; PJ fit, person-job fit; WFC, work-family conflict; FWC, family work conflict; JP, job performance. The AVE for each variable is shown in bold Italics. *P < 0.05, **P < 0.005, ***P < 0.001.

### Common Method Bias

In this study, we assured each respondent the anonymity of the survey and emphasized that there were no right or wrong answers, and we asked the respondents to answer each question as honestly as possible. Since self-report questionnaires may cause common method variance issues, the Harman single-factor test was conducted (Podsakoff et al., 2012). While there is no rule of thumb to indicate how much variance is acceptable, the customary heuristic is to set the threshold to 50%. In this study, no single factor emerged or accounted for the majority of the variance, and the cumulative explained variance of the first factor was 32.367%. The result revealed that there was no evidence of common method bias. However, Podsakoff et al. (2003) recommend using the unmeasured latent method construct (ULMC) method to identify CMV, which is more rigorous than the Harman single-factor test. The ULMC method verifies CMV by specifying factor loadings from a method factor to any or all other items in a model suspected of being contaminated by CMV (Richardson et al., 2009). The results showed that the average substantive variance was 0.5667, and that the average method variance was 0.0199. The ratio was 28:1, which was large enough, and that most of the method factor loadings were insignificant. Therefore, CMV is not a serious concern in this research.

### Structural Model Estimation and Hypotheses Testing

**FIGURE 2:**
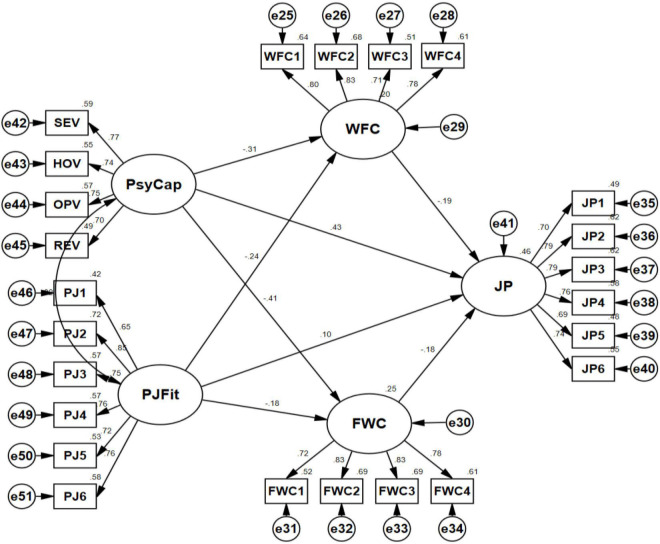
Final structural model.

As the reliability and validity of the full measurement model had been verified, the structural model was evaluated and the hypotheses were tested in the next step. After statistical analysis, fit indices from the structural model demonstrated that the proposed structural model was satisfactory. As shown in Figure 2 and Table 4, there was a significant and negative association between PsyCap and WFC (β = −0.31, *p* < 0.001), and between PsyCap and FWC (β = −0.41, *p* < 0.001), which supported hypotheses 1 and 2. Besides, PJ fit was also negatively correlated with WFC (β = −0.24, *p* < 0.001) and FWC (β = −0.18, p = 0.004); thus, hypotheses 3 and 4 were supported. In addition, both WFC (β = −0.19, *p* = 0.002) and FWC (β = −0.18, *p* = 0.005) had a significant and negative correlation with JP, which supported hypotheses 5 and 6. In addition, there was a significant and positive relationship between PsyCap and JP (β = 0.43, *p* < 0.001), which verified hypotheses 7. However, as presented in Figure 2 and Table 4, PJ fit could not significantly influence JP (β = 0.1, *p* = 0.093); thus, hypothesis 8 was not supported.

**TABLE 4:** Results of the hypothesis test.

Hypotheses	Path	Relation	Parameter estimate	*T*-value	*P*-value	Hypotheses results
H1	PsyCap – > WFC	negative	−0.31	−4.51	***	Supported
H2	PsyCap – > FWC	negative	−0.41	−5.68	***	Supported
H3	PJ fit – > WFC	negative	−0.24	−3.57	***	Supported
H4	PJ fit – > FWC	negative	−0.18	−2.88	0.004	Supported
H5	WFC – > JP	negative	−0.19	−3.05	0.002	Supported
H6	FWC – > JP	negative	−0.18	−2.83	0.005	Supported
H7	PsyCap – > JP	positive	0.43	5.60	***	Supported
H8	PJ fit– > JP	positive	0.10	1.68	0.093	Not Supported

PsyCap, psychological capital; WFC, work-family conflict; FWC, family work conflict; JP, job performance. *P < 0.05, **P < 0.005, and ***P < 0.001.

As can be seen in Table 5, to identify the mediating role of conflict in the work–family interface, bias-corrected and percentile bootstrapping methods were utilized (Preacher and Hayes, 2008). If the bootstrapped CI does not include zero, it means that the mediating effect is different from zero. In this research, 95% bias-corrected CI and 95% percentile CI were estimated with 2,000 bootstrapped samples. To summarize, the results showed that both WFC and FWC significantly and partially mediated the effect of PsyCap on JP. Therefore, hypotheses 9 and 10 were supported. Because of the above-mentioned result that PJ fit could not significantly influence JP, WFC and FWC significantly and fully mediated the impact of PJ fit on JP. That is, hypotheses 11 and 12 were also supported.

**TABLE 5:** Results of the mediation analysis.

			Bias-corrected 95% CI		Percentile 95% CI		
Effects	Path	Beta	Lower	Upper	Lower	Upper	*P*-value
Direct Effects	PsyCap – > JP	0.43	0.265	0.566	0.272	0.569	0.001
Direct Effects	PJ fit– > JP	0.10	-0.022	0.245	-0.031	0.237	0.143
Indirect Effects	PsyCap– > WFC – > JP	0.06 0.07	0.016 0.016	0.112 0.123	0.014 0.019	0.110 0.124	0.009 0.017
Indirect Effects	PJ fit– > WFC – > JP	0.04 0.03	0.009 0.003	0.110 0.084	0.006 0.002	0.101 0.081	0.005 0.033
Total Effects	PsyCap – > JP	0.56	0.409	0.672	0.421	0.681	0.001
Total Effects	PJ fit– > JP	0.18	0.069	0.292	0.065	0.290	0.002

*PsyCap, psychological capital; WFC, work-family conflict; FWC, family work conflict; JP, job performance.*

### Moderating Effects and Hypotheses Testing

In this study, multi-group structural equation modeling was conducted to examine the differences in strengths of structural relationships by assessing the moderating variable effects (Byrne, 2001). The target of multi-group analysis is to verify whether the path coefficients for the relationships between conflicts in the work–family interface and JP were equal in the married and unmarried groups (Yan et al., 2021a). In the moderation tests, the data were categorized into two subgroups according to marital status, namely, 195 married employees and 117 unmarried employees. A two-step method was utilized to conduct a multi-group comparison test. First of all, the appropriate structural parameters are constrained to be equal across the groups to generate an estimated covariance matrix for each subgroup and an overall χ^2^ value for the sets of sub-models as part of a single structural system (Suki, 2014). Second, a second χ^2^ value with fewer degrees of freedom is acquired by removing parameter equality constraints (Suki, 2014). Moderating effects are examined by assessing whether there exist significant differences between the two χ^2^ values. According to Brockman and Morgan (2003), if the change in χ^2^ value is statistically significant, the null hypothesis of parameter invariance is rejected; thus, a moderating effect is verified to exist. As demonstrated in Table 6, Δχ^2^/Δ*df* = 9.98 and *P* < 0.001; there were significant differences between the married and unmarried models. Thus, marital status is a moderator for the relationships mentioned above, which supported hypotheses 13 and 14.

**TABLE 6:** Chi-square value and degree of freedom for the constrained and unconstrained models.

Model	χ ^2^	*df*	Δχ ^2^	Δ*df*	*P-*value
Unconstrained	1275.616	729	19.965	2	<0.001
Constrained	1295.581	731			

*χ^2^, Chi-square; df, degree of freedom.*

Furthermore, Table 7 shows the results of the multi-group comparison test between the married group and the unmarried group with regard to WFC, FWC, and JP. As we can see, for the married group, both WFC and FWC have strong and negative impacts on JP, but for the unmarried employees, WFC can moderately and negatively influence JP. However, there is no significant correlation between FWC and JP.

**TABLE 7:** Results of the multi-group comparison test.

	Married Group			Unmarried Group		
Path	Estimate	S.E.	C.R	Estimate	S.E.	C.R.
WFC → JP	−0.829***	0.174	−4.751	−0.146*	0.073	−2.011
FWC → JP	−0.242***	0.062	−3.917	−0.168	0.113	−1.485

*WFC, work-family conflict; FWC, family work conflict; JP, job performance.*

*P < 0.05, **P < 0.005, and ***P < 0.001.

## Discussion and Conclusion

### Discussion

Employing the COR theory and the congruence theory as theoretical underpinnings, this research proposed and tested a research model to deal with the question of whether PsyCap and PJ fit were linked to JP *via* WFC and FWC. Such relationship was examined based on the data collected from flight attendants who currently work in Air Asia and Malaysia Airlines System Berhad (MAB) in Malaysia. It’s worth noting that the current empirical research has made several important contributions to the existing knowledge.

The findings demonstrated that PsyCap and PJ fit significantly and negatively influenced WFC and FWC, which was consistent with the COR theory (Hobfoll, 2002). According to the COR theory, PsyCap and PJ fit as personal resources can mitigate employees’ conflicts in the work–family interface. Specifically, employees who possess high PsyCap and PJ fit can manage work and family roles effectively and prevent themselves from encountering WFC and FWC. These findings are also in agreement with several previous studies (Wang et al., 2012; Karatepe and Karadas, 2014; Kan and Yu, 2016; Lestari, 2020). However, prior literature is mainly linked to the relationship between one antecedent and two directions of work-family conflicts, and they have not assessed the effects of PsyCap and PJ fit on WFC and FWC simultaneously in Malaysian hospitality industry.

Another critical contribution of this study is the finding that flight attendants with a high level of PsyCap display better performance. That’s because PsyCap can motivate accumulation of personal resources, and individuals with higher PsyCap have confidence in completing tasks successfully and persevere when confronting adversity and challenges. This result also has empirical support from limited empirical studies in the existing literature (Chen, 2015; Guido et al., 2018; Choi et al., 2019). Unlike what we have expected, the results of this study did not confirm the significant and positive relationship between PJ fit and JP. As a matter of fact, O’Neill and Follmer’s (2020) meta-analysis has shown that PJ fit only had a modest correlation with JP (*r* = 0.2). That is to say, the relationship between PJ fit and JP is not as strong and steady as we have anticipated. In addition, Lauver and Kristof-Brown (2001) have proved that PJ fit is not significantly related to task performance in their study. Echoing Lauver and Kristof-Brown (2001) research, Greguras and Diefendorff (2009) pointed out that it was worth noting that the correlation between PJ fit and JP was not significant. The inconsistency in results across studies might be explicated by the omission of important variables (Edwards et al., 1991; Lee and Chu, 2017). Our results suggested that WFC and FWC were intervening variables that assisted to explain the processes through which PJ fit related to JP. As such, it appears that PJ fit is a distal predictor of JP, and that this effect may not be examined in bivariate tests (Shrout and Bolger, 2002). As stressed by Shrout and Bolger (2002): “…for distal processes, the usual bivariate tests of correlation have limited power, it is recommended that the mediation analysis proceeds on the basis of the strength of the theoretical arguments rather than on the basis of the statistical test of X on Y” (p. 430). Hence, the introduction of WFC and FWC as intervening variables makes sense in this study.

This study also adds to the existing knowledge by assessing the effect of the two directions of work-family conflicts on JP simultaneously, which has not been examined before, especially in the hospitality sector. According to the COR Theory, when individuals are faced with high WFC or FWC, they will have to invest extra efforts to deal with both job and family stress, which reduces the amount of available resources for individuals and leads to poor performance (Netemeyer et al., 2005). This finding is congruent with the COR theory and the congruence theory. However, Soomro et al.’s (2018) recent study found that WFC had a significant and positive effect on employee performance (B = 0.1; *P* < 0.01), which was contrary to the results of most relevant studies. They explained that young workers might regard long working hours as a beneficial challenge that can contribute to their life goals. Therefore, from that viewpoint, WFC had a positive correlation with JP.

By examining the moderating effect of marital status, this research enriches extant findings on moderators with which effects of WFC and FWC on JP can be explained in more detail. According to the analysis results, for the married group, both WFC and FWC had strong and negative impacts on JP. However, for the unmarried group, WFC only moderately influenced JP, and even FWC had no significant impact on JP. The reason is that on the one hand, married employees always have more family role responsibilities (Michel et al., 2011). Accordingly, when they are confronted with antecedents, such as working time demands or other work domain stressors, they tend to view these antecedents as conflicts with their family lives compared with employees who are single (Michel et al., 2011). On the other hand, although marital status can be viewed as a type of valued resource based on the COR theory (Grandey and Cropanzano, 1999), if the resources consumed by family responsibilities are much greater than those brought by marital relationship, the married flight attendants will definitely face more WFC and FWC and further affect their job performance.

### Implication

There are also important practical implications emerging from the current research. First, the results demonstrated that PsyCap and PJ fit could decrease conflicts in the work–family interface and promote JP. Thus, rigorous selective procedures for recruiting new employees who possess a high level of PsyCap and PJ fit are supposed to be implemented by human resource departments in hospitality organizations. Second, the results also advocated that the management of hospitality organizations should establish and maintain a family friendly working environment. For example, airline companies can invite employees’ family members to parties that are organized by management and listen to their options and voice to reduce employees’ conflict between family and work roles. Third, more integrated programs that include counseling programs, employee assistance programs, and so on are strongly recommended to get rid of hospitality employees’ stress and promote a support system so that such employees can maintain their psychological resources well. These integrated programs can also give them suggestions on how to balance difficulties between the work and family domains (Karatepe and Magaji, 2008). Once hospitality employees understand their managers’ expectations within the management of work and family roles, the relationship between the two parties may be strengthened on the basis of trust (Karatepe and Magaji, 2008).

### Limitations

Despite this study having made some significant contributions to existing knowledge, we must acknowledge that there are still some limitations. First, the sample of the research was confined to two airline companies in Malaysia, and it is difficult to generalize the results to the whole hospitality industry in Malaysia. Accordingly, future studies can conduct comparative analysis of other airlines or other industries. Second, when assessing mediation, we used a sequential design that has been considered to be a better alternative than cross-sectional design in recent studies. However, in fact, sequential design is only a compromise between cross-sectional and longitudinal designs (Mitchell and Maxwell, 2013; Yan et al., 2021b). Although sequential design incorporates time into a model, it has only one measurement for each X, M, and Y (namely, predictor, mediator, and criterion variables) (Mitchell and Maxwell, 2013). In addition, Maxwell et al. (2011) found that sequential design was also poor in estimating longitudinal mediation parameters. However, sequential design is better than cross-sectional design in estimating indirect effects under partial mediations. Therefore, the direction of deviation in sequential design is generally more predictable than that in cross-sectional design (Maxwell et al., 2011). Maxwell et al. (2007) have emphasized the need to utilize longitudinal design when assessing longitudinal mediation repeatedly, since cross-sectional and sequential designs of the MacArthur method may exaggerate or underestimate direct and indirect effects. In future studies, without the impact of COVID-19, thus, the longitudinal design will be a remedy to avoid such a limitation. Third, this study tested the hypotheses based on self-report data. Although we believe that any potential bias was not a serious problem in our study, we suggest that collecting data from multiple sources in future studies should be implemented in order to minimize this potential problem. Lastly, this study utilized important individual psychological determinants (PsyCap and PJ fit). Future empirical studies should try to investigate other more individual psychological determinants that can affect WFC, FWC, and JP in hospitality or other sectors.

### Conclusion

Employing the COR theory and the congruence theory, this article proposes and verifies the process mechanism of PsyCap and PJ fit influencing JP. By spotlighting the importance of PsyCap and PJ fit in mitigating conflicts in the work-family interface, the study brings more insight for managers into critical individual determinants to relieve flight attendants’ WFC and FWC. What is more, in the context of Malaysian airlines, due to irregular working schedules and psychological stress, flight attendants have to encounter WFC and FWC, which negatively influence JP to a great extent. In addition, married flight attendants are more vulnerable to WFC and FWC and, hence, are more likely to have reduced job performance. The findings demonstrate that more integrated training programs that are associated with PsyCap and PJ fit should be widely popularized. As a result, flight attendants who have received such training are capable of avoiding loss of valuable resources as much as possible. If such employees can maintain a high level of individual psychological resources, they will relieve WFC and FWC effectively so as to advance their JP in the hospitality industry.

## Data Availability Statement

The raw data supporting the conclusions of this article will be made available by the authors, without undue reservation.

## Ethics Statement

The studies involving human participants were reviewed and approved by the Ethical Committee of Qingdao Vocational and Technical College of Hotel Management, Universiti Sains Malaysia and University Putra Malaysia Ethics Committee. The patients/participants provided their written informed consent to participate in this study. The studies followed the Helsinki Declaration with regard to informed consent and human rights, and the treatment of humans in research.

## Author Contributions

ZY performed the data analyses and wrote the manuscript. NB contributed significantly to literature review. ZM contributed to theoretical model and discussion of the study. WC was responsible for reviewing the data and data analyses. All authors contributed to the article and approved the submitted version.

## Conflict of Interest

The authors declare that the research was conducted in the absence of any commercial or financial relationships that could be construed as a potential conflict of interest.

## Publisher’s Note

All claims expressed in this article are solely those of the authors and do not necessarily represent those of their affiliated organizations, or those of the publisher, the editors and the reviewers. Any product that may be evaluated in this article, or claim that may be made by its manufacturer, is not guaranteed or endorsed by the publisher.
